# Supplementary material for: Postprandial Metabolic Effects of Fiber Mixes Revealed by in vivo Stable Isotope Labeling in Humans
Source: Metabolites. 2019 May 7;9(5):91. doi: 10.3390/metabo9050091 (PMC6571904; doi:10.3390/metabo9050091)
Supplement: Supplementary file 1 [file metabolites-09-00091-s001.pdf]

Supplements

Supplementary methods S1: Metabolic network model

$$\frac{dPyr}{dt} = k_1 \cdot c_{Glc} + k_{2b} \cdot L + k_5 \cdot c_{Ala} - (k_{2f} + k_3 + k_{d,Pyr}) \cdot c_{Pyr}$$
$$\frac{dCit}{dt} = k_3 \cdot c_{Pyr} - (k_4 + k_{d,Cit}) \cdot c_{Cit}$$
$$\frac{dLac}{dt} = k_{2f} \cdot c_{Pyr} - (k_{2b} + k_{d,Lac}) \cdot c_{Lac} \quad (3)$$
$$\frac{dGlu}{dt} = k_4 \cdot c_{Cit} - k_{d,Glu} \cdot c_{Glu}$$

where c represent the metabolite concentrations (pyruvate M3 (excluded from the fitting process due to low quality of pyruvate measurement), citrate_M2, lactate_M3, glutamate_M2, glucose_M6 and alanine_M3), k represent the rates of the network and k_d represents the outgoing fluxes. Glucose M6 and alanine M3 were interpolated from data and treated as input to the model (Fig. 7).

The model was fitted to individual subject data in two scenarios. Firstly, assuming that the rate constants in the model were not changed by the interventions, the model was fitted to the control, GG2 and GG4 data simultaneously. The quality of the fit here serves as a baseline, referred to as Model 1 [1] in the following, to compare the results from the other scenario to. In the second scenario, one of the six rate constants was assumed to be influenced by the intervention and thus takes different values for the control, GG2 and GG4 in the fitting procedure (referred to as Model 2 [2] to Model 7 [7] in the following). Please note that the simulated quantities are in principle concentration in cells, which are assumed to be proportional to the blood compartment from which the samples were collected. Therefore four scaling factors, connecting cellular levels to blood levels, were included as free parameters in the fitting procedure. The fitting procedure was guided by minimizing the root mean square (RMS) difference between model simulation (y) and the measured data points ($\hat{y}$).

$$RMS = \sqrt{\sum_{i=1}^{n} \frac{(y_i - \hat{y}_i)^2}{\sigma_i^2}} \quad (4)$$

where σ denotes the standard deviation of the measured data point, and n denotes the number of data points.

A differential evolution based global optimizer was employed for fitting. We allow a large parameter space for the rate constants while the scaling factors are limited in their physiological range. Each optimization task was repeated 50 times, among which the best result was used to calculate the Akaike information criterion with correction (AICc)[1]◎ for model comparison (Eq. 5). The AICc evaluates model quality based on both quality of fitting and the number of free parameters. 2 units larger in AICc means 0.368 times as probable as the model having smaller AICc. $AICc = 2P + RMS^2 + N\log(2\pi) + 2\frac{P(P+1)}{N-P-1}$ (5) where P is the number of fitted parameters, N is the number of data points.

Supplementary Table S1: Results for glucose, insulin and incretin responses (Mean values and percentage differences; 95% confidence intervals vs. control)**[2]**⚜

		GG2			GG4		
	Control	Mean	% Difference	CI	Mean	% Difference	CI
Glucose AUC_{0-120}	148.9	128.4	-13.8	-27.3, 2.3	110.4	-25.9	-37.8, -11.7
Insulin AUC_{0-120}	2322.4	1941.6	-16.4	-27.9, 3.1	1787.7	-23.0	-33.8, -10.5
Insulin AUC_{0-2400}	3541.8	3008.6	-15.1	-24.1, -4.9	2749.7	-22.4	-30.8, -12.9
GIP AUC_{0-120}	2592.9	2437.8	-6.0	-16.3, 5.6	2323.9	-10.4	-20.2, 0.7
GIP AUC_{0-240}	4862.5	4693.3	-3.5	-13.4, 7.6	4568.2	-6.1	-15.7, 4.7
GLP-1 AUC_{0-120}	1579.2	1513.9	-4.1	-15.6, 8.9	1553.1	-1.7	-13.4, 11.7
GLP-1 AUC_{0-240}	3136.6	3057.6	-2.5	-12.0, 8.0	3062.8	-2.4	-11.9, 8.2

Supplementary Table S2: Single metabolite model – Computed appearance rates for the three conditions (Ctrl, GG2 and GG4) and all 12 subjects separately

Interventior	Subject	Glc_M6	Lac_M3	Ala_M3	Cit_M2	Glu_M2	Gly_M2	Ser_M3	Glu_M5	Gln_M5	Val_M5	Tyr_M9	Ile_M6	Lys_M6	Thr_M4
Ctrl	S01	0.268625	0.0113015	0.00261828	0.00071489	0.00066921	0.00283942	0.00441484	0.0045482	0.00518692	0.0040232	0.00633906	0.00367466	0.00453214	0.00376696
Ctrl	S02	0.0220079	0.0020506	0.00029516	0.00016284	0.00045391	0.0005323	0.00027139	0.00075082	0.00030813	0.00018462	0.00030654	0.00078227	0.00092534	0.00031617
Ctrl	S03	0.0492555	0.00464128	0.00155975	0.00045729	0.00147616	0.00182907	0.00312067	0.00342361	0.00436252	0.0021863	0.00417276	0.00328131	0.459228	0.00249909
Ctrl	S04	0.0421099	0.004998	0.0012249	0.00017307	0.00029756	0.00095313	0.00044609	0.00168713	0.00144763	0.0005681	0.00199683	0.00193874	0.00050656	0.00095608
Ctrl	S05	0.0795471	0.00403656	0.00113356	0.00069206	0.00019899	0.00183711	0.00454317	0.00308448	0.00343227	0.00255536	0.00408834	0.00258602	0.00658707	0.00341798
Ctrl	S06	0.0546088	0.00534517	0.00052673	0.00014183	0.00051134	0.00066988	0.00017879	0.00208816	0.00267133	0.00129069	0.00278842	0.00124845	0.00223881	0.00169152
Ctrl	S07	0.0452823	0.00457272	0.00112152	0.00052634	0.00055371	0.00149617	0.00102719	0.00328338	0.00424853	0.00194819	0.00315311	0.00179443	0.00185311	0.00071461
Ctrl	S08	0.0241981	0.00286814	0.00061433	6.2577E-05	0.00021888	0.00052084	6.7447E-05	0.00106885	0.00108619	0.00037835	0.0010788	0.00109779	0.464777	0.00011667
Ctrl	S09	0.0607875	0.00519604	0.00095851	0.00019047	0.00014227	0.0021632	0.00179497	0.00365228	0.00290094	0.00288798	0.00219588	0.00190966	0.00129303	0.00208373
Ctrl	S10	0.0530479	0.00595327	0.00102628	0.00029978	0.00034266	0.00155552	0.00090885	0.00252158	0.00236119	0.00109332	0.00223033	0.0024288	0.00051738	0.00214582
Ctrl	S11	0.0432863	0.00499588	0.00089157	0.00013682	0.00036397	0.00108035	0.00072725	0.00078908	0.00129312	0.00054138	5.1195E-05	0.00137689	0.00240477	0.00052367
Ctrl	S12	0.0788965	0.00903065	0.00138995	0.00028877	0.00040642	0.00187535	0.00267765	0.00495653	0.0045104	0.00425731	0.00132404	0.0013879	0.00104941	0.00161107
GG2	S01	0.0528563	0.00513484	0.00120524	0.00014722	0.00019911	0.00247408	0.00206673	0.0030818	0.00288032	0.00163171	0.00384732	0.00420306	0.0022965	0.00250763
GG2	S02	0.031756	0.00438085	0.00041516	0.00021262	0.00014513	0.0006041	7.0677E-05	0.00064937	0.00111651	0.00029106	0.00053544	0.00142905	0.00035556	0.00043765
GG2	S03	0.0376186	0.00392976	0.00132798	0.00033531	0.00195955	0.00157328	0.00395088	0.00276272	0.003458	0.00213801	0.00434527	0.00383415	0.424564	0.0037635
GG2	S04	0.0252275	0.00480754	0.00150668	0.00036558	0.00025906	0.00120423	0.00110366	0.00230673	0.00113931	0.00115126	0.00222358	0.0015448	0.00307399	0.00155678
GG2	S05	0.0481041	0.00418359	0.00075497	6.9001E-05	0.00043147	0.000909	0.00197806	0.00162498	0.00168995	0.00073433	0.00249184	0.00213883	0.0055775	0.00090319
GG2	S06	0.022548	0.00373774	0.00064729	0.00011304	0.00017965	0.00034904	0.00089172	0.00059398	0.00059286	0.00145497	0.00075721	0.00011367	0.00307309	0.00030782
GG2	S07	0.0276112	0.00456184	0.00100637	0.00022401	0.00027783	0.00180036	0.00153612	0.00319586	0.00366134	0.00191381	0.0025956	0.00290862	0.00086757	0.00269314
GG2	S08	0.0334162	0.00249044	0.0005327	0.00022011	0.00028996	0.00596406	0.00019287	0.00127686	0.00099241	0.00078472	3.6288E-05	0.00107109	3.1155E-05	0.0029542
GG2	S09	0.0456506	0.00592601	0.00134883	0.00024862	0.00033242	0.00214376	0.00174603	0.00255419	0.00369518	0.00464702	0.001877	0.00213603	0.0044092	0.00477235
GG2	S10	0.0632677	0.00372459	0.00118073	0.00017389	0.00057016	0.0014246	0.00214468	0.00245035	0.00230061	0.00153006	0.00251255	0.00386818	0.00779165	0.00276549
GG2	S11	0.0209897	0.00301203	0.00040733	0.00017386	0.00022319	0.00042839	0.00016984	0.0002943	0.00030491	0.00017473	2.318E-05	0.00061444	0.00185203	6.7603E-05
GG2	S12	0.0631225	0.00547805	0.00111746	0.00035061	0.00015684	0.00174481	0.00187989	0.00355094	0.003362	0.00226594	0.00160613	0.00165873	0.00275417	0.00063827
GG4	S01	0.108865	0.00537761	0.0011309	0.00046307	0.00081809	0.00060365	0.0012851	0.00402703	0.00409072	0.0024666	0.00412457	0.00220272	0.00386024	0.00265048
GG4	S02	0.0213215	0.00286915	0.00045113	0.00010029	0.00030738	0.0004381	0.00029147	0.00079137	0.00057408	0.00019445	0.00018096	0.00022741	0.00025999	0.00048087
GG4	S03	0.0217949	0.00304545	0.00073096	0.00367369	0.00036181	0.00077964	0.00149259	0.001631	0.0025496	0.0006476	0.00146474	0.00284872	0.00081283	0.00113357
GG4	S04	0.0230384	0.00412267	0.00099924	7.7282E-05	0.00023231	0.00057336	0.00049947	0.00059535	0.0019478	0.00042836	0.00084505	0.0005532	0.00126505	0.00097704
GG4	S05	0.0670327	0.0042731	0.00112305	0.00581881	0.00055382	0.00194855	0.00343408	0.00262068	0.00437114	0.00628237	0.00468632	0.00335661	0.00320733	0.00340245
GG4	S06	0.021839	0.00248159	0.00042018	0.00010763	0.00019731	0.00059582	0.0002397	0.00071321	0.00036761	0.00046144	0.00065644	0.00023191	0.00145741	0.00014798
GG4	S07	0.0310165	0.00533275	0.00080353	0.00036018	0.00036258	0.00104998	0.00057665	0.00275989	0.00235137	0.00186314	0.00218499	0.0038362	0.00011986	0.00634524
GG4	S08	0.0252632	0.0046199	0.00071122	0.00014244	0.00031781	0.0029031	0.00190613	0.00180852	0.00210653	0.00096966	0.00023983	0.00242503	0.160922	0.00170818
GG4	S09	0.0379984	0.00482021	0.00095575	0.00014079	0.00026812	0.00159325	0.00024311	0.00302389	0.00304261	0.0033764	0.0031363	0.00072961	0.00162905	0.00182645
GG4	S10	0.0314977	0.00330763	0.00089354	0.00039307	0.00032729	0.00137093	0.00074484	0.00170924	0.00152888	0.00099249	0.00212019	0.00281125	0.00304704	0.00133004
GG4	S11	0.0167067	0.0021402	0.0004186	0.00014078	6.0493E-05	0.00063319	0.00028686	0.00022775	0.00022516	0.00012984	0.00012211	0.0893092	0.00095727	0.00023539
GG4	S12	0.0214161	0.00439013	0.00065387	0.00017925	0.0001368	0.00034202	6.7906E-05	0.00091628	0.00034144	0.00027464	0.00041258	0.00033964	0.00101247	0.139306

Supplementary Figure S1: Postprandial effect of wheat bread intake – Heatmap of significantly altered metabolites over time (Median of all subjects, Baseline correction, zScore Normalization)

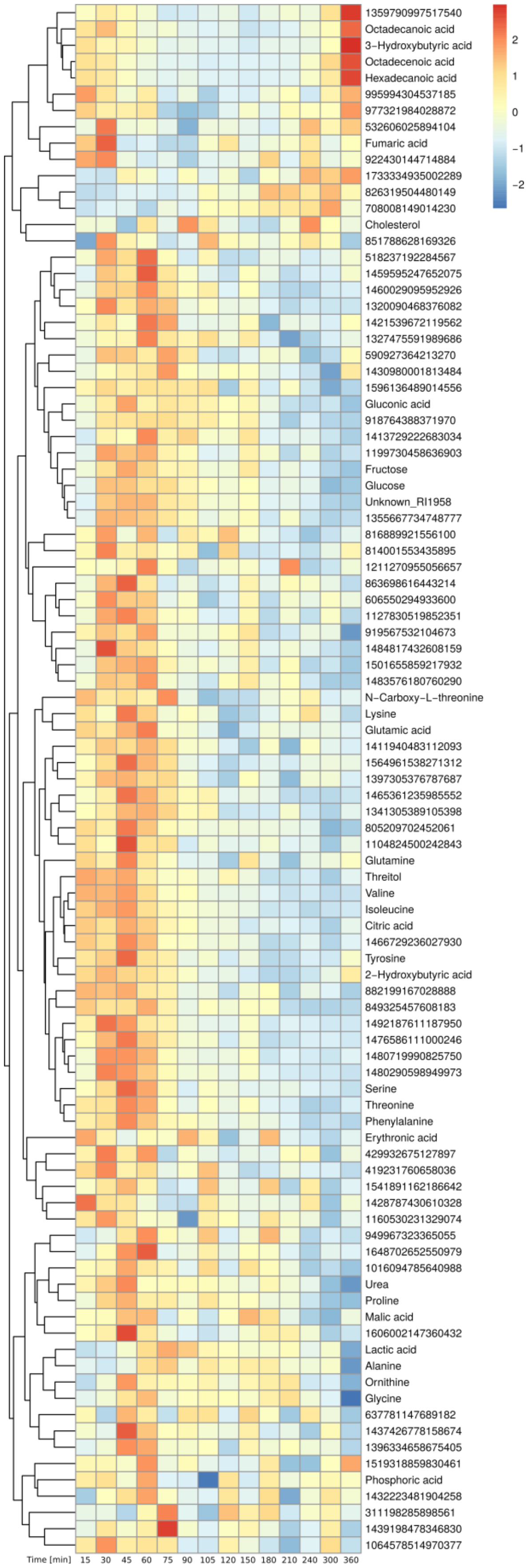

Supplementary Figure S2: Time-resolved metabolite levels after wheat bread intake – Line graphs of medians of all subjects with median average deviation

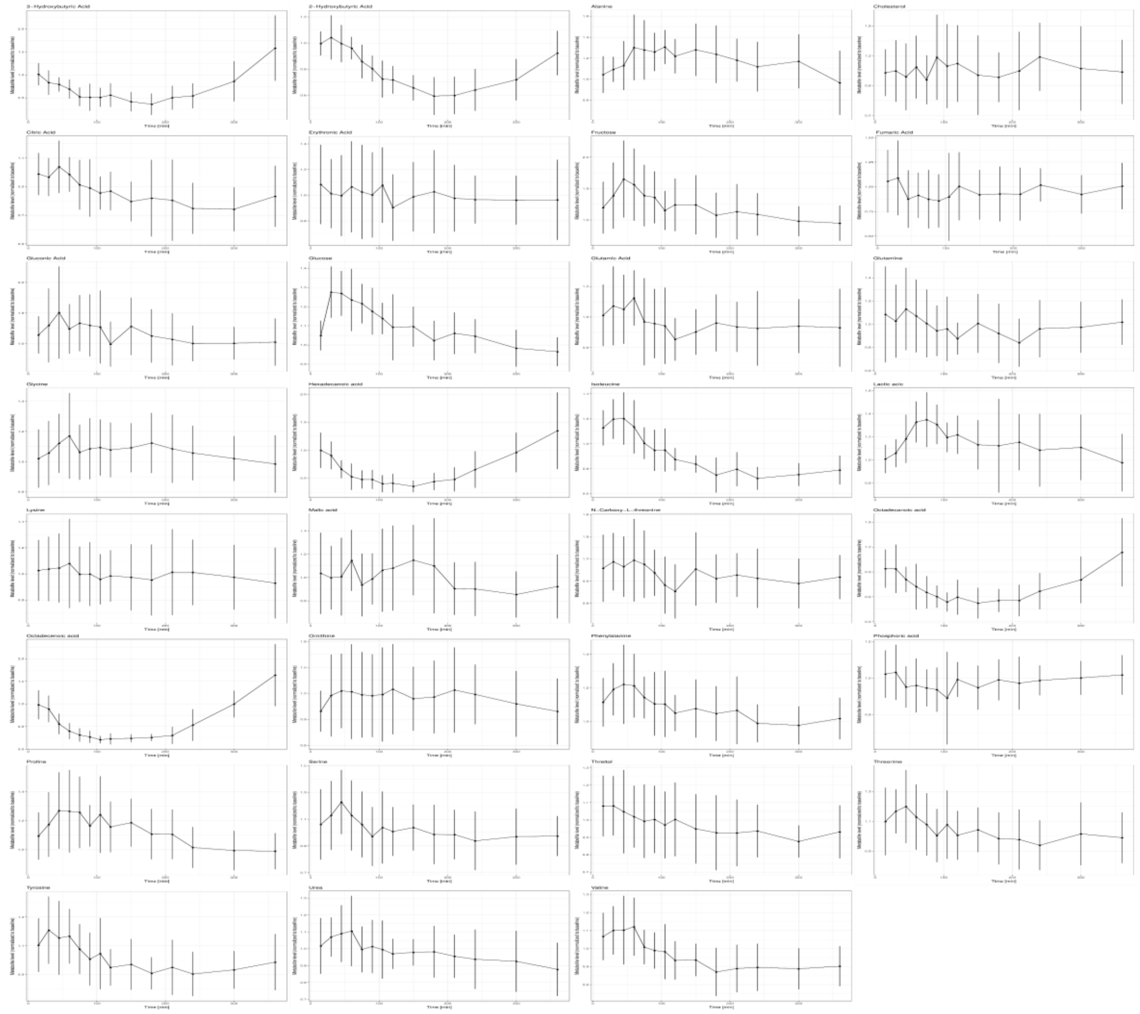

Supplementary Figure S3: ^{13}C-enrichment profile over time upon intervention for glutamine M2 – due to low enrichment, reliable calculations were omitted and negative values were obtained (Red – Control, Green – GG2, Blue – GG4; Average of MI abundance in % ± standard error of 12 subjects; upper box – starch-derived metabolites, lower box – metabolites of mixed origin)

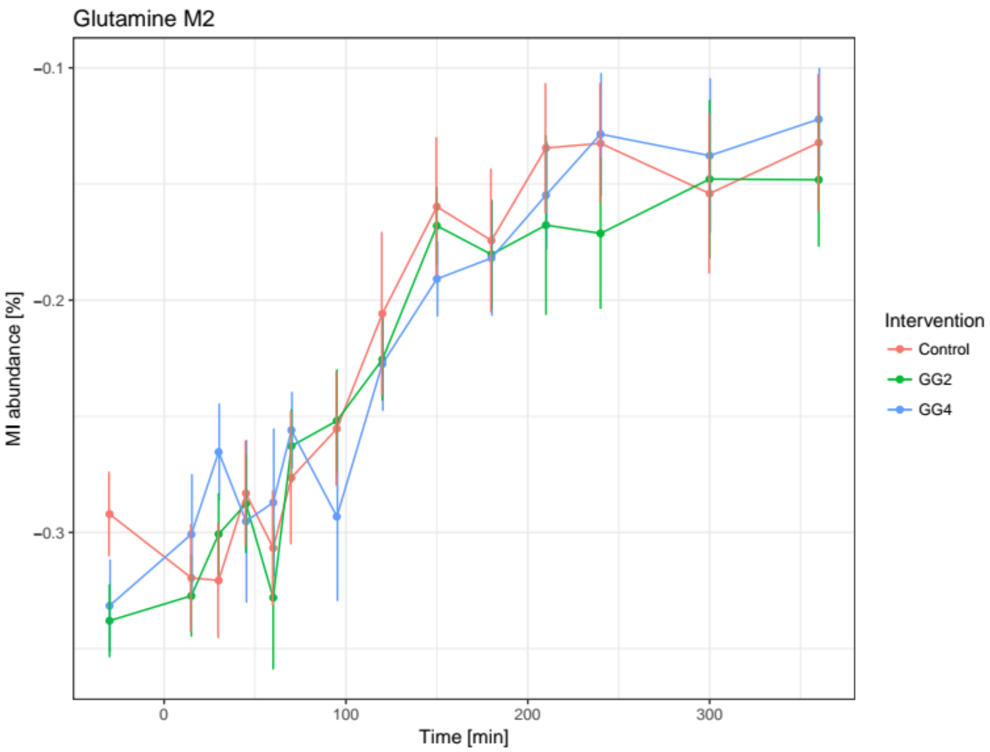

Supplementary Figure S4: Significantly altered metabolite level profiles upon intervention – Fructose, 1355667734748777 (putative Gluconolactone), Gluconic acid, Unknown_1958 (putative Allonic acid gamma lactone), Cholesterol and palmitic acid, stearic acid, octadecenoic acid, 3-hydroxybutyric acid and 2-hydroxybutyric acid

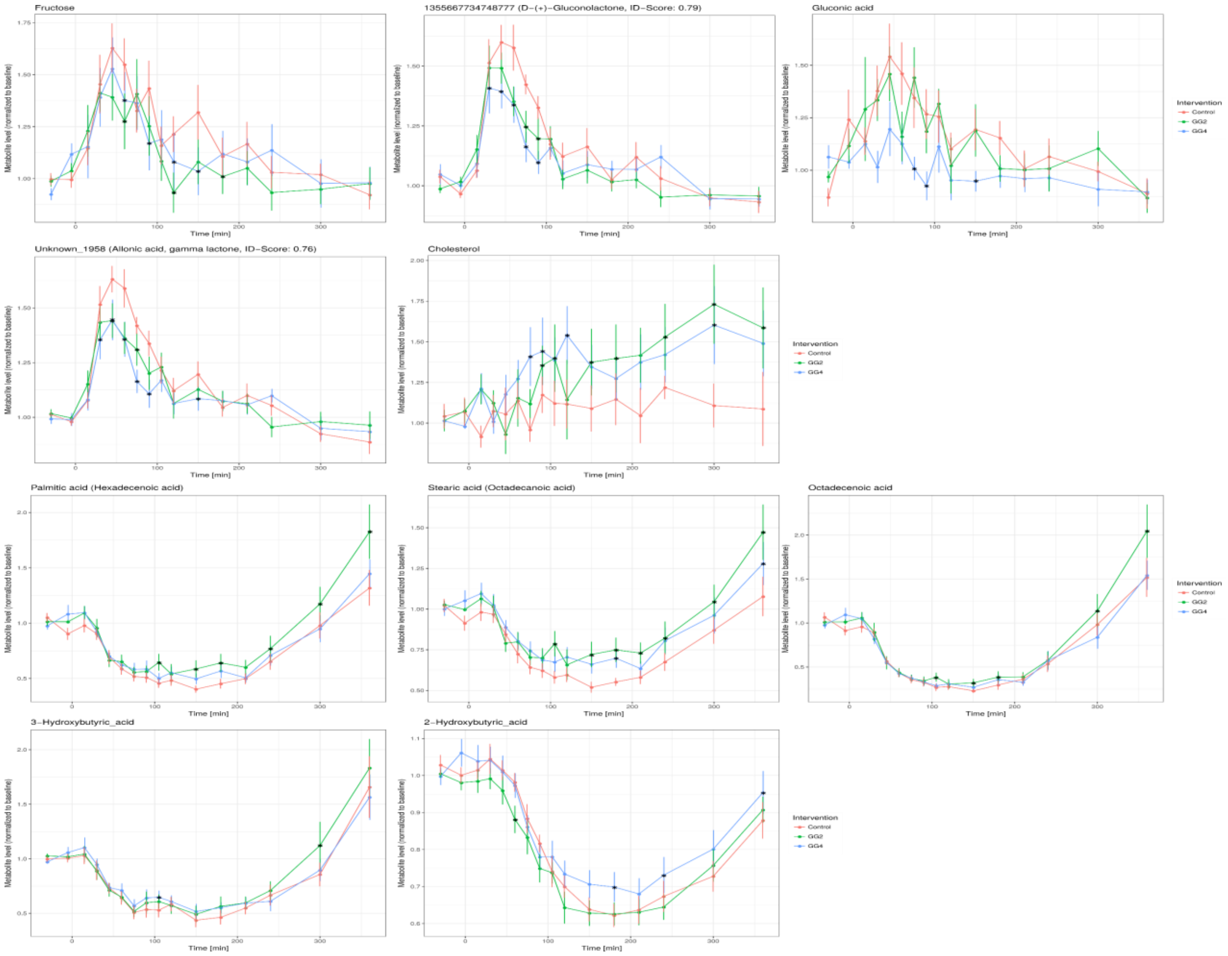

Supplementary Figure S5: Response curves of ^{13}C-enrichment profiles over time upon intervention for protein-derived metabolites – glutamic acid M5, glutamine M5, valine M5, tyrosine M9, isoleucine M6 (Red – Control, Green – GG2, Blue – GG4; Average of MI abundance in % ± standard error of 12 subjects)

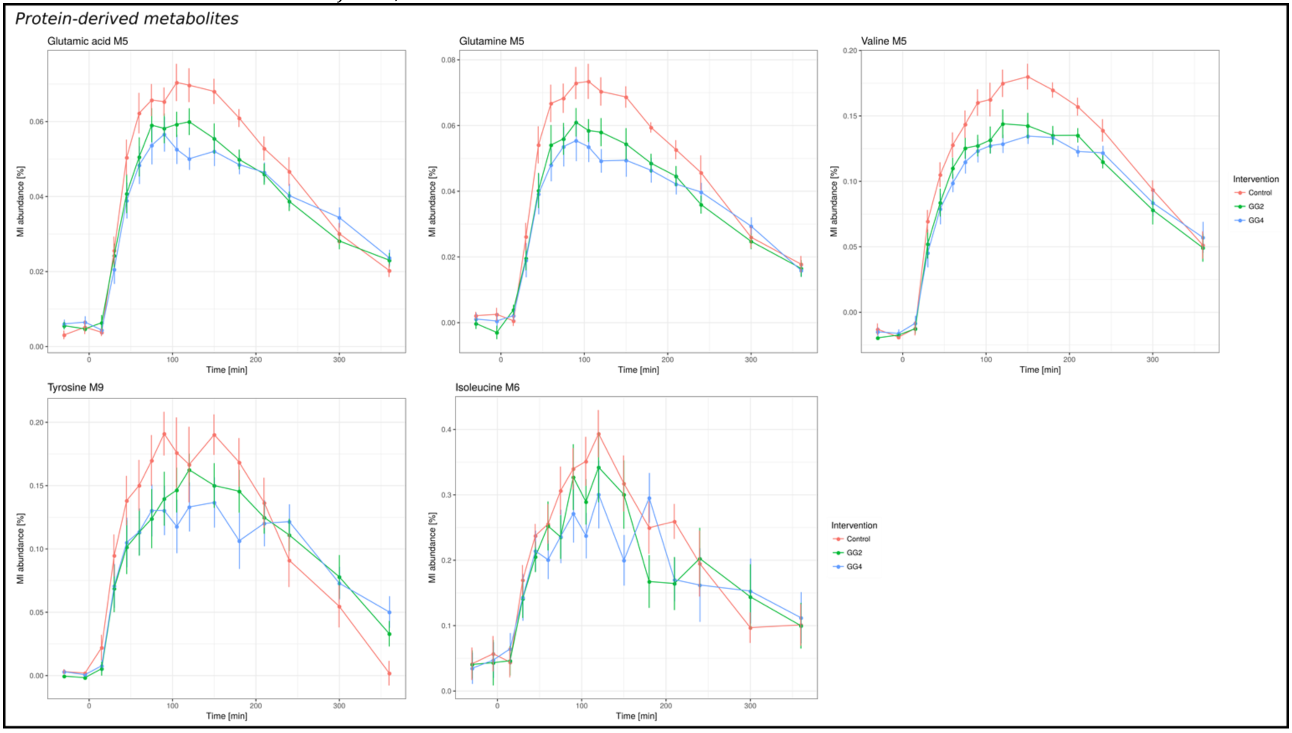

Supplementary Figure S6: Metabolic network model – a) Network model connecting the metabolites glucose, pyruvate, lactate, alanine, citrate and glutamate where model [1] represents the fixed rates model and [2]-[7] represent the flexible rates models where the number indicates the respective flexible rates (e.g. model [2] – rate k_3 flexible); b) AICcs calculated for the 7 different models (indexed in a) and normalized to the AICc obtained for Model 1 (all rates fixed) for all 12 subjects separately, blue dashed line indicates significantly decreased AICc compared to Model 1.

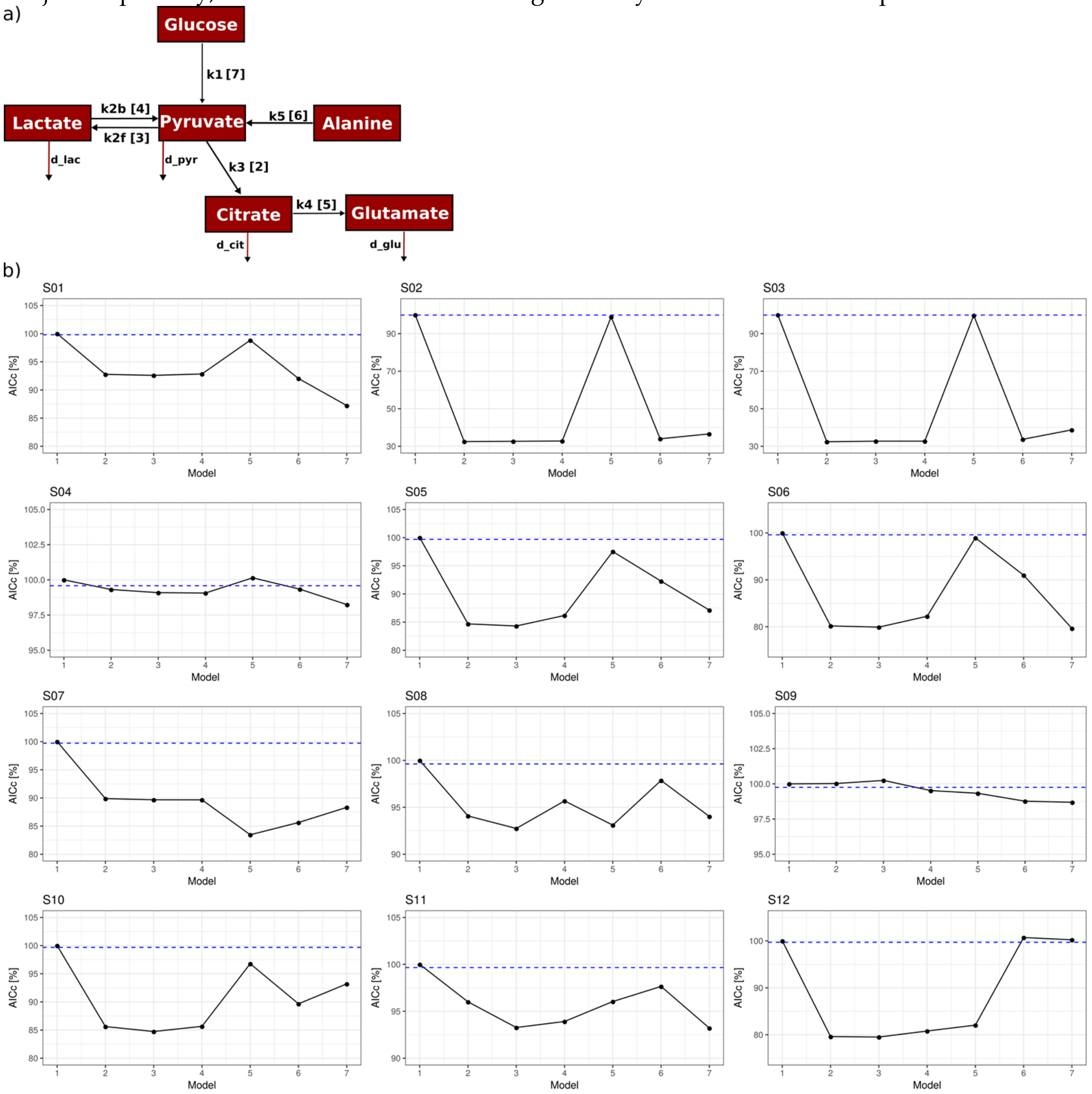

References

[1] Cavanaugh, J.E., 1997. Unifying the derivations for the Akaike and corrected Akaike information criteria. Statistics & Probability Letters 33(2): 201–8, Doi: 10.1016/S0167-7152(96)00128-9.

[2] Boers, H.M., Van Dijk, T.H., Hiemstra, H., Hoogenraad, A.R., Mela, D.J., Peters, H.P.F., et al., 2017. Effect of fibre additions to flatbread flour mixes on glucose kinetics: A randomised controlled. British Journal of Nutrition 118(10): 777–87, Doi: 10.1017/S0007114517002781.
